# The Psychological Dimensions of Dieting: A Two-Phase Study on Body Appreciation, Nutritional Awareness and Mental Well-Being

**DOI:** 10.3390/nu18091405

**Published:** 2026-04-29

**Authors:** Paula Sophia Cozma, Lóránd Dénes, Zsuzsánna Simon-Szabó

**Affiliations:** 1 Faculty of Medicine, George Emil Palade University of Medicine, Pharmacy, Science and Technology of Târgu Mureș, 540142 Târgu Mureș, Romania; 2 Anatomy and Embryology Department, Faculty of Medicine, George Emil Palade University of Medicine, Pharmacy, Science and Technology of Târgu Mureș, 540142 Târgu Mureș, Romania; 3 Pathophysiology Department, Faculty of Medicine, George Emil Palade University of Medicine, Pharmacy, Science and Technology of Târgu Mureș, 540142 Târgu Mureș, Romania

**Keywords:** dieting, body appreciation, nutritional awareness, mental health, eating disorder screening, psychoeducational intervention

## Abstract

Background/Objectives: Dieting is a widespread behavior that is associated with psychological distress, body dissatisfaction, and eating disorders. Recent research suggests that a body-positive attitude and mindful approach to eating may influence individuals’ experiences with dieting; however, their combined role has been insufficiently explored. Methods: A two-phase study was conducted among voluntary adults using online data collection. In Phase 1, a cross-sectional survey was completed by 180 participants (71.7% women), assessing dieting behavior, body appreciation, nutritional awareness, psychological distress, well-being, and eating disorders. Correlation analyses, group comparisons, and regression models were performed. In Phase 2, 90 participants entered the pilot and received a brief psychoeducational digital material promoting mindful eating and positive body image. The follow-up assessment was completed by 59, after one month of engagement. Results: Body appreciation and nutritional awareness were positively associated with mental well-being and inversely related to psychological distress (*p* < 0.001 for all) and to eating disorder screening scores (*p* < 0.001 and *p* = 0.046, respectively). More frequent dieting was associated with lower body appreciation (*p* < 0.001). According to the observed pattern of correlations, body appreciation may play a role in the relationship between dieting and psychological distress. In the intervention phase, greater engagement with the psychoeducational material was associated with higher reported levels of nutritional awareness (*p* = 0.003) and greater perceived body awareness (*p* = 0.026) at follow-up; however, due to the exploratory design, findings are preliminary. Conclusions: The results suggest that dieting, as a behavior, may be embedded in broader psychological processes that include body-related attitudes and nutritional awareness. Taking these factors into account may have potential implications for preventive measures aimed at promoting healthier dietary habits, a more positive relationship with one’s body, and mental well-being.

## 1. Introduction

Weight-loss diets are particularly popular in Western societies, with a prevalence ranging from 21 to 56% among women and from 6 to 25% among men [1]. In addition, numerous studies focusing on restrained and weight-control behaviors describe similarities between eating habits and dieting patterns among normal-weight and overweight individuals [2]. However, dieting itself is a heterogeneous construct that includes various types of dietary practices [3,4]. It has become a widespread practice across all age groups and demographic clusters, driven not only by weight loss goals, but also by the desire to maintain health, prevent and manage diseases, and achieve aesthetic goals [2]. Although dieting has traditionally been defined primarily as an adjustment of dietary behavior and a form of eating restriction [1,5], it is increasingly recognized as a psychological process of great significance. Several studies suggest that eating-related behaviors are linked to body image [6] and broader indicators of mental health, including depression, anxiety, eating disorders (EDs) and psychological well-being, as reported in community-based and general population samples. Importantly, the psychological and behavioral consequences of dieting may vary depending on the type and underlying motivation of the dietary practices involved [2].

Both negative and positive psychological states are relevant within the broader framework of mental health, especially when examining eating-related behavior. Mental health is a psychological state characterized, among others, by emotional well-being, relative freedom from anxiety and disability-causing symptoms, as well as the ability to cope with everyday stress [7]. Psychological distress is a concept that covers a range of common clinical and subclinical psychological states, from stress to depressive symptoms and anxiety, which can impair mental health and well-being [8]. In contrast, mental well-being is a positive dimension of mental functioning and refers to effective functioning in daily life, marked by feeling of satisfaction and a sense of purpose [7]. These parameters are especially important in terms of eating behavior, as psychological distress is associated with maladaptive eating habits, while an increased sense of mental well-being is related to healthier behavior regulation and lifestyle [9].

EDs represent an interrelated but distinct area of mental health and belong to a group of psychiatric disorders characterized by chronic disturbance of eating behavior and body image. These conditions exist within a spectrum; therefore, early detection is essential [10].

Body image holds a central position within this context. It is defined as a complex, multidimensional construct, incorporating both positive and negative perceptions, thoughts, feelings, beliefs, and behaviors to one’s body. Previous research has predominantly been focused on its negative dimension, particularly body dissatisfaction and its consequences. This attitude has been linked to EDs, unhealthy eating behaviors, an increased risk of developing EDs, as well as anxiety and depression [11,12,13]. In contrast, a positive body image does not merely signify the absence of a negative body image but also reflects a flexible and holistic perspective that is associated with greater mental well-being and milder depression symptoms [14,15].

One of the most well-established dimensions of positive body image is body appreciation, which is characterized by acceptance, respect, and gratitude toward the body. Higher levels of body appreciation have been associated with lower ED symptomatology, more adaptive and greater psychological well-being [16,17]. These findings suggest that body appreciation may play a central role in both nutrition-related outcomes and broader psychological context, potentially functioning as a protective factor.

In addition to body-related behavior, awareness-based aspects of nutrition also play a key role in understanding eating behavior. Mindful eating and awareness during mealtimes focus on recognizing the body’s signals, such as emotional and physical hunger, signs of satiety, and sustaining attention throughout the mealtime [18]. Increasing evidence suggests that mindful eating may be an effective method for reducing unhealthy eating habits, as it emphasizes the internal, psychological aspects of eating [19]. In contrast, nutritional awareness refers more to conscious attention to the content of one’s diet than to the experience of the eating process itself [18,20]. This includes deliberate food choices, which are shaped by an individual’s nutritional knowledge and perceived health effects [21]. Greater nutritional awareness has been associated with more favorable dietary habits.

Given the growing concerns about the link between dieting, eating habits, and their psychological consequences, evidence is emerging regarding preventive and educational interventions aimed at raising awareness and promoting healthier behavioral patterns. Research suggests that effective educational interventions require a shift from a traditional information-based perspective to the principles of behavioral science [22,23]. Thus, psychoeducational and behavior-oriented interventions can contribute to improvements in body image, well-being, and eating behavior, and may also help reduce the risk of EDs, particularly among outpatients [11,23]. Even low-intensity, self-administered methods, including digital formats, have demonstrated potential benefits. These approaches are particularly important from a nutrition perspective in public health, where widely accessible tools are essential for promoting sustainable health changes. Practical advantages of the digital intervention content include time efficiency and the ability to reach people living in remote areas or those who would otherwise be less likely to request help [11].

Despite growing interest in the psychological processes associated with dieting, previous studies have typically examined body image, nutritional awareness, and mental health either separately or with varying degrees of integration [14,24,25,26]. Although some research has begun to address these areas jointly, their interrelationships within a comprehensive framework remain only partially understood. Furthermore, evidence regarding the potential effects of brief, low-intensity digital psychoeducational interventions on changes in body image and eating-related processes is still emerging [11,22].

To address this gap, a two-phase study was conducted that combined a cross-sectional survey with an experimental digital intervention. The primary aim of the study was to examine the psychological dimensions of dieting in an adult population. Dieting was conceptualized as a heterogeneous, self-reported construct, and the analyses focused on the associations between dieting-related behavior, nutritional awareness, body appreciation, and key mental health outcomes, including mental well-being, psychological distress and the likely case of EDs.

The goal of the first phase was to explore how dieting and nutritional awareness are correlated with both positive and negative dimensions of mental health. It was hypothesized that frequent dieting and lower levels of nutritional awareness would be associated with poorer body appreciation and less favorable mental health outcomes, while greater body appreciation and higher levels of nutritional awareness would be associated with higher well-being, lower psychological distress, and a lower screen score for eating disorders.

In the second phase, the exploratory component examined the potential short-term associations related to a brief digital psychoeducational intervention designed to promote mindful eating and a positive body image. It was expected that greater engagement with the material would be linked to higher self-reported changes in body appreciation and eating-related attitudes.

## 2. Materials and Methods

### 2.1. Study Design

A two-phase study design was applied to examine the psychological aspects of dieting in the adult population. Data collection was conducted online in each phase. Phase 1 consisted of a cross-sectional survey in which volunteer participants completed a series of self-administered questionnaires that assessed psychological and dietary variables at a given point in time. Phase 2 was a pilot study that involved a brief psychoeducational intervention and a post-intervention follow-up assessment conducted on a subgroup of participants.

### 2.2. Enrollment Procedures and Methods

Participants were recruited through online distribution platforms and social media channels. Convenience sampling was applied. Enrollment and data collection were conducted via Google Forms. The questionnaire was disseminated primarily through Facebook and Instagram. It was also shared in over 50 online groups related to dieting in order to reach a broader and more diverse audience. Recruitment was not actively monitored beyond tracking the number of completed responses.

The eligibility criteria included: participants ≥ 18 years of age, willing to complete the online questionnaire independently, and providing informed consent. Anyone with a psychiatric diagnosis, receiving psychotropic medication in the past four weeks, and pregnant at the beginning of the study was excluded. The sample size was guided by feasibility considerations; given the study design, no formal sample size calculation was performed.

The study was conducted in accordance with the Declaration of Helsinki and obtained approval from the Institutional Research Ethics Committee of George Emil Palade University of Medicine, Pharmacy, Science and Technology of Târgu Mureș.

In Phase 1, participants completed an anonymous online questionnaire assessing dietary behaviors, psychological variables, and screening test scores. All Phase 1 participants were invited to take part in Phase 2.

In Phase 2, only a portion of the participants took part. Due to the exploratory and pilot nature of the study, no prior power analysis was conducted, nor was a control group included, as the primary objective was to assess feasibility, acceptability, and preliminary changes associated with the intervention rather than to establish causal relationships. Participants who agreed to continue received a psychoeducational digital material in PDF format via email. For this purpose, email addresses were collected separately from the survey data and were not linked to participants’ responses to ensure anonymity.

The material was developed through the professional collaboration of a psychologist and a registered dietitian. It was based on the principles of psychoeducation, mindful eating approaches and positive body image, supported by relevant scientific literature (see Supplementary Material File S1 for details; corresponding references are included in the main reference list). It combined theoretical concepts related to body image, the psychological aspects of dieting, motivation-related processes, and the principles of awareness-based eating with practical exercises focused on self-reflection, goal setting, mindful eating, self-affirmation, and gratitude toward the body. The overall structure was designed to help participants develop a healthier body image and more mindful eating habits.

Participants were instructed to read the material, complete the included exercises, and apply the recommendations in their daily lives over a one-month period. This timeframe was considered sufficient to engage with the content and record any short-term changes observed. All participants were invited to complete a follow-up questionnaire at the end of the study period and received several reminders; however, completion was voluntary, and dropout was not systematically controlled. Engagement with the material was not directly monitored; instead, participants reported their level of use and perceived application of the material in the follow-up questionnaire.

#### 2.2.1. Phase 1 Measures

All participants provided their age and gender. Their body mass index (BMI) was calculated based on self-reported height and weight.

The dietary habits were evaluated using a set of study-specific closed-ended, semi-closed-ended and Likert-type self-report items covering diet-related attitudes and behaviors. Nutritional awareness was measured using a single Likert-type question that assessed the extent to which participants consciously considered their dietary choices. Responses were recorded on a 5-point scale ranging from 1 (not at all) to 5 (very much), with higher scores indicating greater perceived nutritional awareness. Since it was assessed using a single, non-validated item, internal consistency was not applicable, and the measure captures a general, self-perceived aspect of nutritional awareness.

Dieting behavior was evaluated as a broad construct, encompassing diverse dietary practices. It was assessed via questions regarding dieting history and frequency of attempts (never, once, multiple times), type of diet followed (multiple-choice options, including medical diets, low-calorie diets, ketogenic diets, intermittent fasting, and others), and primary motivation for dieting (e.g., health-related, aesthetic reasons).

Positive body image was assessed using the Body Appreciation Scale-2 (BAS-2), which is a free, validated tool that measures an individual’s acceptance, respect, and appreciation of their body. The responses were listed on a 5-point scale in ascending order; the results were averaged to obtain the overall score, with higher scores indicating higher levels of body appreciation [13,17]. The questionnaire has a unidimensional structure, excellent internal consistency (α ≈ 0.93–0.94), high test–retest reliability (ICC ≈ 0.90), and strong construct validity for both college and community samples.

Main symptoms of psychological distress were assessed using the Patient Health Questionnaire-4 (PHQ-4), an ultra-brief screening tool for depression (2 questions) and anxiety (2 questions), which has demonstrated a good internal consistency (α = 0.85) and strong construct validity across general and primary care samples. The 4 statements are scored on a Likert scale ranging from 0 to 3, and the total score is calculated by summing the responses to all statements (the maximum score is 12 points). A score between 0 and 2 indicates the absence of psychological distress. A score between 3 and 5 indicates mild distress, between 6 and 8 indicates moderate distress, and between 9 and 12 indicates severe distress. The scale is freely available to the public [27,28].

Mental health assessment was conducted using the World Health Organization’s 5-item Well-Being Index (WHO-5). This short and widely used self-assessment scale measures subjective well-being, which is rated on a Likert scale ranging from 0 to 5, so the raw score ranges from 0 (lack of well-being) to 25 (maximum well-being). The raw scores were multiplied by 4 to obtain a percentage scale ranging from 0 to 100. It has been proven that the scale is a reliable and valid instrument in various populations [29,30].

The risk of EDs was assessed using the validated SCOFF questionnaire, which consists of five yes/no questions. The acronym SCOFF is derived from the keywords in the questions: “Sick,” “Control,” “One stone,” “Fat,” and “Food.” This brief self-assessment questionnaire covers the essential characteristics of anorexia nervosa and bulimia. Each “yes” answer is worth one point, and a total score exceeding two points indicates a likelihood of anorexia nervosa or bulimia. The questionnaire is not intended for diagnostic purposes; rather, it is commonly used as a brief screening tool and has been applied in both clinical and non-clinical populations [31,32].

All instruments were administered in Romanian and Hungarian. Where available, previously translated versions of the standardized questionnaires (BAS-2, PHQ-4, WHO-5, SCOFF) were used; otherwise, translations were prepared for the purposes of the study. Minor linguistic adaptations were made to ensure clarity and comprehensibility. The study was not intended to validate the translated instruments, but rather to apply them in an exploratory context.

#### 2.2.2. Phase 2 Measures

The follow-up questionnaire assessed subjective evaluation of the material (Processing Index, 5 items), its perceived usefulness, engagement with its content, as well as observed changes in nutrition and body image-related attitudes or behaviors (Change Index, 7 items). The assessment consisted of closed, semi-closed and open questions tailored to the study. Both indexes were derived as the means of the items on the four-point Likert scale, where higher scores indicated a more positive assessment and greater change.

### 2.3. Statistical Analysis

Statistical analyses were performed using standard statistical software. Descriptive statistics were used to summarize the sample characteristics and the study variables. Due to the non-normal distribution of several variables, non-parametric statistical tests were used (Spearman, Mann–Whitney U, Kruskal–Wallis).

Multiple linear regression analyses were conducted to examine the relationship between the key variables using the standard enter method. Despite some variables deviating from normality, linear regression was considered appropriate due to its robustness to moderate violations of this assumption. Ordinal variables measured on multi-point scales were treated as continuous to facilitate their inclusion in regression models.

Assumptions of linear regression were tested. Residuals were examined for normality, and no substantial deviations were found. No multicollinearity was detected. Homoscedasticity was assessed visually using residual plots, and no major violations were observed.

Gender differences in the frequency of dieting were examined using nonparametric tests, and gender was included as an additional covariate in separate regression analyses to control its potential role in the observed associations.

Statistical significance was set at *p* < 0.05.

In Phase 1, correlation analyses and group comparison tests were conducted to explore the primary outcomes, the association between eating-related behavior and psychological variables. Regression analyses were performed to examine associations between key variables and psychological outcomes.

In Phase 2, correlation matrices and multiple linear regression analysis were used to examine associations between changes in attitudes and material evaluation. Group comparisons were also performed according to the level of participant engagement.

## 3. Results

### 3.1. Cross-Sectional Findings—Phase 1

#### 3.1.1. Sample Characteristics

A total of 210 people participated in the survey, and after applying the eligibility criteria, data from 180 participants were included in the study. The majority of respondents were women (71.1%), and the largest age group was 18–25 years (35.6%). Based on BMI categories, 41.1% of participants were of normal weight, 5.0% were underweight, and the rest were overweight or obese, with an average BMI of 25.7 ± 5.64 kg/m^2^. In terms of previous dieting experience, 70.6% reported having dieted before, and more than half had tried dieting more than once. No significant gender differences were found in dieting frequency. The most common motivation for dieting was health-related reasons (39.3%) and aesthetic reasons (26.7%), while the remaining responses (33.8%) fell into the “other” category, representing less frequently reported motivations. Participants reported following various types of diets, which were categorized into four main groups: intermittent fasting, hypo- and hypercaloric diets, exclusion-based diets (characterized by the exclusion or substantial reduction in specific food groups, e.g., ketogenic diet, sugar-free diet, gluten-free diets) and medically prescribed diets. The categories were not mutually exclusive, as participants could report multiple types of diets. Detailed descriptive statistics, including psychological variables, are presented in Table 1.

#### 3.1.2. Correlational Analyses

Spearman’s rank correlations showed significant associations between key variables in the study (Table 2). Dieting frequency was positively correlated with higher SCOFF scores (*p* < 0.001) and negatively correlated with body appreciation (*p* < 0.001). Moreover, nutritional awareness showed a positive correlation with body appreciation (*p* = 0.003) and well-being (*p* < 0.001), and a negative correlation with psychological distress (*p* < 0.001) and the likelihood of eating disorder (*p* = 0.001). In addition, body appreciation (BAS-2) showed a positive correlation with well-being (*p* < 0.001), and a negative correlation with psychological distress (*p* < 0.001) and higher SCOFF scores (*p* < 0.001).

#### 3.1.3. Regression Models

Multiple linear regression analyses were performed to examine associations with the primary outcomes. The frequency of dieting (*p* < 0.001) and nutritional awareness (*p* = 0.006) were significantly associated with body appreciation. The PHQ-4 score was significantly linked to BAS-2 score (*p* < 0.001), nutritional awareness (*p* < 0.001) and BMI (*p* = 0.019). Similarly, the WHO-5 score showed significant associations with BAS-2 score (*p* < 0.001) and nutritional awareness (*p* < 0.001). Finally, the SCOFF was significantly related to BMI (*p* < 0.001), frequency of dieting (*p* = 0.011), and nutritional awareness (*p* = 0.004). The regression models accounted for a modest to moderate proportion of variance across the outcomes (see Table 3 for detailed regression coefficients). No multicollinearity was detected.

The inclusion of gender as an additional covariate did not substantially alter the observed associations, and gender was not significantly associated with any of the outcomes in the regression models.

**Figure 1:**
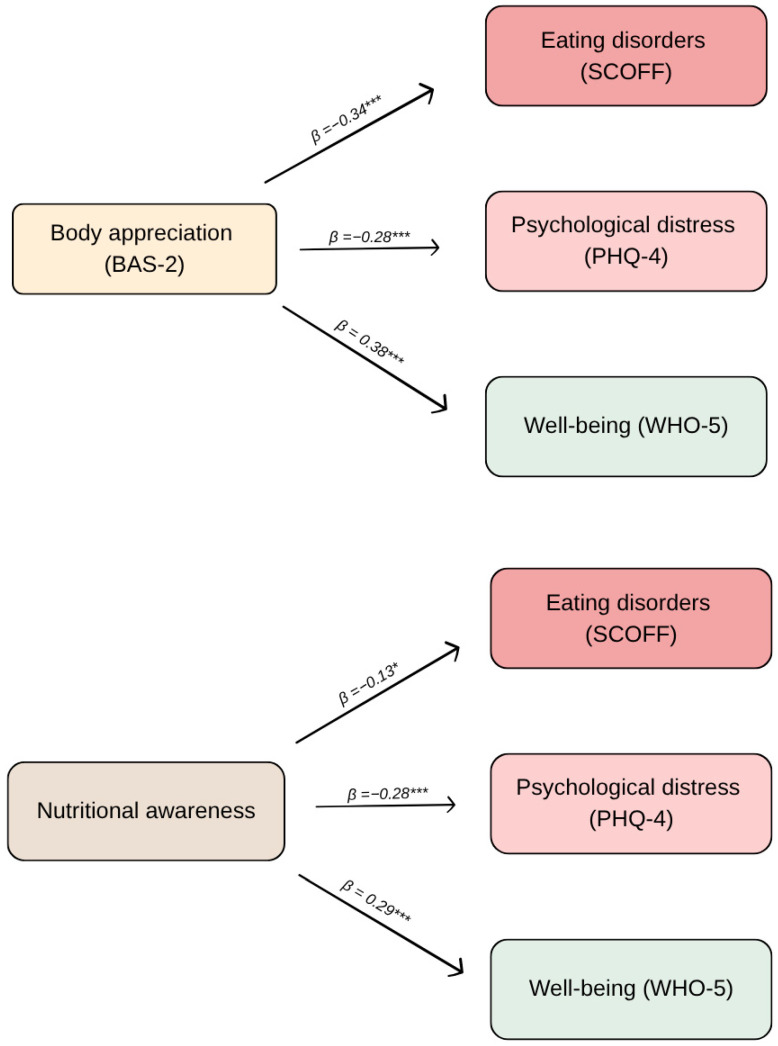
Associations of body appreciation and nutritional awareness with mental health outcomes. Note. Values represent standardized regression coefficients (β) derived from multiple linear regression analyses. Standardized regression coefficients (β) from multiple linear regression models are presented. Arrows indicate statistically significant associations between variables derived from separate models (see Table 3). * *p* < 0.05, *** *p* < 0.001.

The associations are further illustrated in Figure 1, where similar patterns were observed across the mental health outcomes.

#### 3.1.4. Group Comparisons

Participants with a previous history of dieting had lower BAS-2 scores than non-dieters (3.50 vs. 4.02, *p* = 0.002) and higher SCOFF scores (0.98 vs. 0.49, *p* = 0.007). There was no difference in terms of well-being or distress.

A similar pattern was observed across the different diet frequency groups: significant differences were found between non-dieters, one-time dieters, and recurrent dieters (*p* = 0.002 for the BAS-2; *p* = 0.004 for the SCOFF). Recurrent dieters showed lower body appreciation and higher SCOFF scores compared to those who had dieted only once.

Significant differences were also observed among the diet motivation groups (*p* ≤ 0.005); participants who dieted for aesthetic reasons reported lower body appreciation and higher SCOFF scores than those motivated by health reasons.

Across BMI categories, significant differences were also found for body appreciation and the likelihood of EDs (*p* < 0.05). Obese participants had the highest SCOFF scores (1.64) and lower BAS-2 scores (2.95) than normal-weight and overweight participants. Well-being and distress did not differ significantly across the categories.

Differences were also evident across the range of nutritional awareness scores. As nutritional awareness increased, participants reported higher well-being (29.3 vs. 69.6; *p* < 0.001), lower distress (6.50 vs. 1.33; *p* < 0.001), lower likelihood of EDs (0.83 vs. 0.39; *p* < 0.001), and higher body appreciation (3.67 vs. 3.89; *p* = 0.028).

No significant differences were found in the psychological outcomes across different types of dieting (*p* > 0.05).

Detailed results of group comparisons are shown in Table 4A,B.

#### 3.1.5. Pattern of Associations Between Key Variables

**Figure 2:**
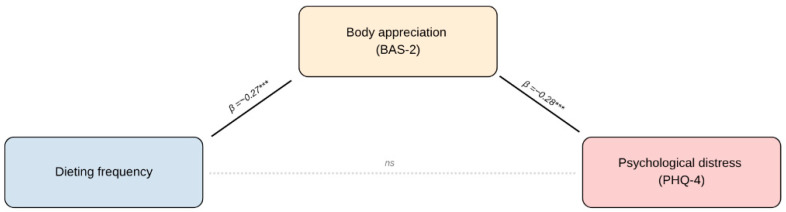
Patterns of association between dieting frequency, psychological distress and body appreciation. Note. Values represent standardized regression coefficients (β). Solid lines denote statistically significant paths, whereas the dashed line indicates a non-significant direct association (ns). *** *p* < 0.001.

The frequency of dieting was significantly associated with body appreciation (β = −0.267, *p* < 0.001). Body appreciation was also significantly associated with psychological distress in the regression model (β ≈ −0.30, *p* < 0.001). In contrast, the association between dieting frequency and distress was not statistically significant (Figure 2).

### 3.2. Interventional Findings—Phase 2

#### 3.2.1. Engagement and Feasibility

Of the 90 individuals who participated in the pilot, 59 completed the follow-up questionnaire. Most of them read the material and completed at least one exercise; nearly half reported that they had applied the recommendations multiple times (Table 5). The mean score for the Processing Index was 3.60 (SD = 0.37), while the mean of the Change Index was 3.07 (SD = 0.51), both measured on a 0–4 scale.

#### 3.2.2. Determinants of Perceived Change

The Processing Index scores showed a positive correlation with the Change Index scores (ρ = 0.481, *p* < 0.001) in the follow-up assessment. Linear regression analysis indicated that the Processing Index was significantly associated with Change Index scores (b = 0.658, *p* < 0.001), accounting for 22.4% of the variance.

Engagement indicators showed varying correlations with perceived change. Literacy level was not correlated with Change Index scores (*p* = 0.688). However, the participants who had completed the exercises scored higher on the Change Index than those who had not (*p* = 0.048). Differences in Change Index scores were also observed between levels of application (χ^2^(2) = 6.22, *p* = 0.045). Active application was also associated with higher nutritional awareness (*p* = 0.003) and greater perceived body awareness (*p* = 0.026) at follow-up.

## 4. Discussion

This two-phase study provides an integrated perspective on the psychological dimensions of dieting and the potential relevance of a brief psychoeducational digital intervention designed to promote awareness-based eating attitudes and a positive relationship with one’s body. The key findings from the cross-sectional phase indicate that body appreciation and nutritional awareness were consistently associated with more favorable mental health outcomes, including greater well-being, lower psychological distress, and a lower likelihood of eating disorders. Consistent with the study’s hypotheses, more frequent dieting and lower levels of nutritional awareness were linked to lower body appreciation, while higher levels of body appreciation and greater nutritional awareness were associated with more favorable psychological outcomes. Furthermore, the findings suggest that, within this sample, dieting may not be understood merely as a behavioral strategy primarily related to weight management, but rather a psychologically embedded process in which body-related attitudes and mindfulness-based eating habits may serve as key regulatory factors. The novelty of this study lies in the integration of dieting, body appreciation and nutritional awareness into a single analytical framework, as well as in the combination of cross-sectional and experimental intervention data.

Phase 1 results indicate that body appreciation plays a key role within the psychological structure of eating-related behaviors. More frequent dieting was consistently associated with poorer body appreciation, even when BMI was included in the model, suggesting that subjective body-related experiences may play a role beyond objective weight status in understanding dieting outcomes. It should be noted, however, that differences across BMI categories were observed at the descriptive level in body appreciation, with lower scores in obese individuals, a pattern consistent with previous research [33,34]. Moreover, higher body appreciation was closely associated with greater psychological well-being, lower levels of distress, and a reduced likelihood of EDs. These findings are consistent with the current view that a positive body image is not merely the absence of dissatisfaction, but a psychological resource on its own [11]. More broadly, the present findings suggest that body appreciation may play a key role in the relationship between dietary behavior and its psychological consequences.

In the present study, the psychometric results yielded a multifaceted picture of mental health by capturing indicators of both positive and negative aspects of functioning. Psychological distress (PHQ-4) and well-being (WHO-5) constitute complementary dimensions of general mental health, while eating disorders (SCOFF) reflect a more specific area of eating-related psychopathology. The results suggest that body-related attitudes and awareness-based eating habits are linked not only to common emotional states but also with domain-specific risk indicators. Their links to both well-being and distress highlight the broader relevance to overall mental health within this sample, while the associations with eating disorder screening scores underline their potential clinical significance. Overall, these findings suggest that the psychological dimensions related to dieting are associated with multiple levels of mental functioning, rather than being limited to individual domains in isolation.

A key finding of the study is the observed pattern of associations linking dieting frequency, body appreciation and psychological distress. Dieting frequency was not directly related to psychological distress, but it was significantly associated with body appreciation, which was in turn related to higher distress. This pattern suggests that the psychological burden associated with dieting-related behaviors assessed in this study may be reflected in body-related attitudes, highlighting their potential role in the relationship between dieting and psychological distress. This distinction may have important conceptual implications, as it shifts the focus from dieting itself to the psychological meaning and context of dieting. This interpretation is consistent with biopsychosocial models of eating behavior that highlight the role of cognitive and emotional processes in shaping health outcomes [35].

In the cross-sectional phase, awareness-based eating models showed complementary patterns. Higher nutritional awareness was consistently associated with more favorable psychological profiles, including higher well-being, lower anxiety and depressive symptomatology, higher body appreciation and reduced likelihood of EDs. These correlations may reflect the more conscious and informed dietary decision-making habits of individuals with greater nutritional awareness, although this finding may be interpreted with caution. Moreover, awareness-oriented approaches may enhance self-regulation by shifting attention from externally driven appearance pressures to internal cues and informed decisions, thereby promoting adaptive behavioral and psychological regulation [19,36,37].

In addition, significant differences were observed regarding dieting frequency, with less favorable psychological outcomes among recurrent dieters. Importantly, in this study, “dieting” was defined as a heterogeneous, self-reported concept that incorporates a range of eating practices (intermittent fasting, calorie-based, exclusion-based, and medically prescribed diets). Within this framework, no significant differences were found across the different types of dieting practices. The divergent trends observed further underscore the fact that not all forms of self-regulation regarding eating, including both frequency and type of dieting, have the same psychological implications.

The underlying motivation for dieting provided further insight. Appearance-motivated dieting was associated with poorer body appreciation and a greater likelihood of EDs compared to health-motivated dieting, suggesting that the intent behind dietary restriction may have a more important role in shaping psychological outcomes than the form of dietary behavior assessed. This observation is consistent with previous evidence that external-focused goals centered on physical appearance are more closely linked to body dissatisfaction and maladaptive eating habits, while health-focused goals reflect more adaptive self-care motivations [2,38]. Notably, a considerable proportion of participants reported motivations that were not covered by the previously defined categories, suggesting that dietary behavior may often be driven by complex or heterogeneous motivations.

Overall, the Phase 1 results indicate an interconnected psychological system in which body-related attitudes and awareness-based eating processes are associated with mental health. Body appreciation was closely linked to well-being and inversely correlated with distress, while nutritional awareness was linked to both positive and negative dimensions of mental health. This integrated pattern may reflect the multidimensional model of mental health, suggesting that multiple psychological processes may be involved simultaneously in domains related to eating.

The results of the intervention phase extend the cross-sectional observations to an applied prevention context. This phase was designed as a pilot, exploratory component to assess the feasibility and preliminary psychological relevance of a low-intensity psychoeducational digital intervention. Such experimental work may be relevant when considering scalable prevention approaches, particularly in non-clinical populations where short and accessible tools are needed [39]. The high level of commitment and favorable subjective ratings indicate that the material was acceptable and feasibly implementable, suggesting its potential viability within this sample as a preventive educational resource. However, it should be considered that the participants who completed the follow-up questionnaire may represent a more motivated subgroup.

Importantly, engagement quality appeared to be more relevant than mere exposure. Reading the material alone was not associated with perceived change, while completing the exercises and actively applying the recommendations was related to higher perceived change. This pattern may reflect a dose–response-like relationship; however, causal conclusions cannot be drawn. The perceived usefulness and comprehensibility of the material were significantly associated with self-reported change, indicating that commitment and perceived relevance may play a key role in low-intensity interventions. This observation is consistent with the educational and behavioral science frameworks, suggesting that information alone may not be sufficient, whereas active processing and personal relevance may contribute to more pronounced change [22,23].

Participants who reported performing the exercises more intensively also reported changes in body awareness and in the attentional processes related to eating at follow-up. Notably, this pattern parallels the central findings of the cross-sectional phase, in which body appreciation and awareness-based eating attitudes were consistently associated with mental health outcomes. Such conceptual convergence between observational and pilot findings may support the potential relevance of awareness-based processes and body-related factors as targets for preventive strategies. Nevertheless, these findings should be interpreted cautiously, as causal conclusions cannot be established. Rather, the consistency between phases may indicate conceptual alignment within the proposed explanatory framework.

The findings may have implications for nutrition practice and public health. Traditional dietary approaches often emphasize calorie control and weight loss while neglecting the psychological determinants of sustainable behavior. The present study suggests that incorporating elements of positive body image and awareness-based eating constructs may be relevant in this context. Digitally available, low-intensity psychoeducational materials may represent accessible and cost-effective tools, especially in public health settings that focus primarily on prevention.

Several limitations warrant consideration. The cross-sectional design of Phase 1 excludes causal inferences and does not allow conclusions about the directionality of the associations. All measurement methods were self-reported, which may contribute to response bias and reporting inaccuracies. In addition, the use of convenience sampling and online recruitment may introduce selection bias, which could potentially limit the representativeness of the sample. Although no significant gender differences were observed, the gender imbalance in the sample, together with the relatively young age distribution, may limit the generalizability of the results. Moreover, nutritional awareness was measured with a single, study-specific item, which may limit the precision and construct validity of this variable. Finally, due to the relatively small number of participants in some BMI categories, subgroup differences should be interpreted with caution, and future studies should further investigate potential differences between weight groups.

In Phase 2, additional limitations are particularly relevant. The relatively small and self-selected sample, the lack of a control group, the reliance on subjective changes and the short follow-up period limit the generalizability and interpretability of the findings. A potential ceiling effect of the material assessment may have further reduced variability. The anonymized design of the study precluded linking responses across phases; therefore, baseline differences and comparisons between completers and dropouts could not be assessed, and self-selection bias cannot be excluded. Consequently, it is not possible to distinguish the effects of the intervention from natural variation or expectancy effects. These results should therefore be considered preliminary and hypothesis-generating rather than confirmatory.

Several strengths of the study are also worth mentioning. The two-phase design allowed for both cross-sectional modeling and preliminary intervention testing within the same conceptual framework. Mental health was assessed multidimensionally, incorporating well-being, psychological distress, and risk of EDs. The inclusion of positive body image and awareness-based eating constructs enabled a more nuanced psychological interpretation that transcended traditional dietary measures. Furthermore, the evaluation of a scalable, low-intensity digital intervention enhances the public health relevance of the findings.

Future research should address these limitations by employing more rigorous methodological designs. Longitudinal and randomized controlled trials are necessary to clarify causal relationships and determine temporal relationships between dietary behavior, body-related attitudes, and mental health status. The involvement of control groups and larger, more diverse samples could improve statistical power and increase the generalizability of findings. In addition, examining potential moderators, such as BMI categories, could refine explanatory models of mental health processes related to dieting. Future research should include objective behavioral indicators in addition to self-report measures to obtain a more comprehensive picture of eating-related processes. Extending follow-up periods may allow for an assessment of the sustainability and practical significance of changes related to the intervention. Digital materials enhanced with interactive and personalized elements may further increase engagement and effectiveness.

## 5. Conclusions

This study contributes to a more integrated understanding of dieting by suggesting that eating behaviors may be conceptualized as a part of a broader psychological system that links body image, nutritional awareness, and mental health. The results indicate that more positive attitudes toward the body and higher levels of nutritional awareness are associated with more favorable psychological outcomes, including greater well-being, lower distress, and a reduced likelihood of EDs.

The exploratory intervention phase suggests that psychoeducational approaches focusing on awareness may be associated with changes in awareness-related attitudes toward eating and the body, although these findings should be interpreted cautiously, given the pilot nature of the study.

Overall, the results highlight the potential relevance of incorporating psychological concepts into the study of dietary behavior. Further research is needed to clarify the nature and direction of these relationships and evaluate the effectiveness of the related interventions.

## Figures and Tables

**Table 1:** Sample characteristics and descriptive statistics of participants (Phase 1).

Variable	Total (N = 180)
**Age group, n (%)**	
18–25 years	64 (35.6%)
26–35 years	33 (18.3%)
36–45 years	22 (12.2%)
46–50 years	35 (19.4%)
**Sex, n (%)**	
Female	129 (71.7%)
Male	51 (28.3%)
**BMI category, n (%)**	
Underweight	9 (5.0%)
Normal weight	74 (41.1%)
Overweight	70 (38.9%)
Obesity	27 (15.0%)
**Dieting history, n (%)**	
Previous dieting	127 (70.6%)
Repeated dieting attempts	101 (56.1%)
**Type of diet, n (%)**	
Intermittent fasting	48 (38.1%)
Hypo- and hypercaloric diets	31 (24.6%)
Exclusion-based diets	37 (29.4%)
Medically prescribed diets	14 (11.0%)
**Motivation for dieting, n (%)**	
Health-related	50 (39.3%)
Aesthetics	34 (26.7%)
Other	43 (33.8%)
**Nutritional awareness, mean ± SD**	3.41 ± 0.95
**Psychological variables, mean ± SD**	
Body appreciation (BAS-2)	3.65 ± 0.98
Psychological distress (PHQ-4)	2.96 ± 2.61
Well-being (WHO-5)	56.9 ± 20.0
Eating disorders (SCOFF)	0.83 ± 1.04
**Risk categories, n (%)**	
PHQ-4 moderate distress	21 (11.7%)
PHQ-4 severe distress	7 (3.9%)
SCOFF medium risk	47 (26.1%)
SCOFF high risk	42 (23.3%)

Note. Values are presented as frequency (n) and percentage (%) or mean ± standard deviation (SD). BAS-2 = Body Appreciation Scale-2; PHQ-4 = Patient Health Questionnaire-4; WHO-5 = World Health Organization Well-Being Index; SCOFF = Sick, Control, One stone, Fat, Food questionnaire.

**Table 2:** Spearman correlations among key study variables.

Variable	1	2	3	4	5	6
1. Body appreciation (BAS-2)	—					
2. Dieting frequency	−0.263 ***	—				
3. Nutritional awareness	0.219 **	ns	—			
4. Psychological distress (PHQ-4)	−0.334 ***	ns	−0.293 ***	—		
5. Well-being (WHO-5)	0.447 ***	ns	0.308 ***	−0.574 ***	—	
6. Eating disorders (SCOFF)	−0.406 ***	0.245 ***	−0.238 **	ns	−0.194 **	—

Note. 1 = Body appreciation (BAS-2); 2 = Dieting frequency; 3 = Nutritional awareness; 4 = Psychological distress (PHQ-4); 5 = Well-being (WHO-5); 6 = Eating disorders (SCOFF). Values represent Spearman’s ρ coefficients. ns = non-significant. ** *p* < 0.01; *** *p* < 0.001.

**Table 3:** Multiple linear regression models for primary outcomes.

Outcome (Dependent Variable)	Independent Variable	Standardized β	95% CI	*p*
**Body appreciation (BAS-2)**	Nutritional awareness	0.203	[0.060, 0.346]	0.006
	Dieting frequency	−0.267	[−0.417, −0.118]	<0.001
	BMI	−0.092	[−0.241, 0.056]	0.222
	Adjusted R^2^ = 0.11			
**Psychological distress (PHQ-4)**	Body appreciation	−0.284	[−0.429, −0.140]	<0.001
	Nutritional awareness	−0.276	[−0.418, −0.135]	<0.001
	Dieting frequency	−0.030	[−0.180, 0.119]	0.068
	BMI	−0.174	[−0.319, −0.029]	0.019
	Adjusted R^2^ = 0.16			
**Well-being (WHO-5)**	Body appreciation	0.377	[0.242, 0.513]	<0.001
	Nutritional awareness	0.293	[0.160, 0.427]	<0.001
	Dieting frequency	−0.051	[−0.192, 0.090]	0.476
	BMI	0.011	[−0.125, 0.148]	0.867
	Adjusted R^2^ = 0.25			
**Eating disorder (SCOFF)**	Body appreciation	−0.336	[−0.469, −0.202]	<0.001
	Nutritional awareness	−0.133	[−0.264, −0.002]	0.046
	Dieting frequency	0.096	[−0.041, 0.235]	0.170
	BMI	0.266	[0.132, 0.400]	<0.001
	Adjusted R^2^ = 0.28			

Note. Values represent standardized regression coefficients (β) with 95% confidence intervals (CIs) from the multiple linear regression models. Adjusted R^2^ values indicate the proportion of variance explained by each model. No multicollinearity detected.

**Table 4:** (**A**). Group differences in psychological outcomes across study variables. (**B**). Group differences in eating disorder screening scores (SCOFF) across study variables.

(A)					
Variable	Outcome	Groups	Mean ± SD	Test Statistic	*p*-Value
Dieting history	BAS-2	Yes vs. No	3.50 ± 1.01 vs. 4.02 ± 0.82	U = 2404	0.002
Dieting frequency	BAS-2	No vs. Once vs. Multiple times	4.02 ± 0.82 vs. 3.77 ± 0.99 vs. 3.43 ± 1.01	χ^2^ = 12.38	0.002
Dieting motivation	BAS-2	Aesthetic vs. Health vs. Other	2.94 ± 0.97 vs. 3.74 ± 0.99 vs. 3.63 ± 0.95	χ^2^ = 13.04	0.005
BMI categories	BAS-2	Underweight vs. Normal vs. Overweight vs. Obese	3.78 ± 0.83 vs. 3.82 ± 0.90 vs. 3.67 ± 1.03 vs. 2.95 ± 1.00	χ^2^ = 13.10	0.011
Nutritional awareness	BAS-2	1–5	3.67 ± 1.21 vs. 3.39 ± 1.03 vs. 3.42 ± 0.94 vs. 3.86 ± 0.96 vs. 3.89 ± 0.96	χ^2^ = 10.80	0.028
	PHQ-4		6.50 ± 3.45 vs. 4.00 ± 2.83 vs. 2.88 ± 2.29 vs. 2.79 ± 2.53 vs. 1.33 ± 1.88	χ^2^ = 22.30	<0.001
	WHO-5		29.3 ± 8.26 vs. 47.1 ± 19.8 vs. 56.5 ± 18.6 vs. 59.5 ± 18.7 vs. 69.6 ± 20.3	χ^2^ = 22.70	<0.001
**(B)**					
**Variable**		**Groups**	**Mean ± SD**	**Test Statistic**	***p*-Value**
Dieting history		Yes vs. No	0.98 ± 1.12 vs. 0.49 ± 0.72	U = 2404	0.007
Dieting frequency		No vs. Once vs. Multiple times	0.49 ± 0.72 vs. 0.65 ± 0.98 vs. 1.07 ± 1.14	χ^2^ = 10.87	0.004
Dieting motivation		Aesthetic vs. Health vs. Other	1.61 ± 1.17 vs. 0.84 ± 1.04 vs. 0.69 ± 1.01	χ^2^ = 14.97	0.005
BMI categories		Underweight vs. Normal vs. Overweight vs. Obese	0.11 ± 0.33 vs. 0.61 ± 0.83 vs. 0.83 ± 1.05 vs. 1.64 ± 1.18	χ^2^ = 24.99	<0.001
Nutritional awareness		1–5	0.83 ± 1.33 vs. 0.91 ± 1.04 vs. 1.28 ± 1.15 vs. 0.56 ± 0.90 vs. 0.39 ± 0.50	χ^2^ = 18.70	<0.001

Note. Values are presented as mean ± standard deviation (SD). Test statistics are reported as Mann–Whitney U for two-group comparisons and χ^2^ values of Kruskal–Wallis tests for multiple groups. Only statistically significant results are presented.

**Table 5:** Phase 2 outcomes: engagement, evaluation, and perceived change.

Domain	Variable	n (%)/Mean ± SD
**Engagement**	Fully read material	48 (81.4%)
	Completed exercises	44 (74.6%)
	Applied recommendations once	22 (37.3%)
	Applied recommendations multiple times	28 (47.5%)
**Material evaluation**	Processing Index	3.60 ± 0.37
**Perceived change**	Change Index	3.07 ± 0.51

Note. Values are presented as n (%) or mean ± standard deviation. The Processing Index reflects clarity, usefulness, and applicability of the material. The Change Index reflects self-perceived changes in eating-related attitudes and body awareness. Higher scores indicate a more positive evaluation and greater perceived change.

## Data Availability

The data presented in this study are available on request from the corresponding author due to privacy and ethical restrictions.

## Supplementary Materials

The following supporting information can be downloaded at https://www.mdpi.com/article/10.3390/nu18091405/s1, File S1: Psychoeducational material used in Phase 2. References [5,13,25,37,40,41,42,43,44,45,46,47,48] are cited in the Supplementary Materials.

## Abbreviations

The following abbreviations are used in this manuscript:

BMI	Body Mass Index
BAS-2	Body Appreciation Scale-2
ED	Eating Disorders
PHQ-4	Patient Health Questionnaire-4
SCOFF	Sick, Control, One stone, Fat, Food questionnaire
WHO-5	World Health Organization’s 5-item Well-Being Index
