# Supplementary material for: The Psychological Dimensions of Dieting: A Two-Phase Study on Body Appreciation, Nutritional Awareness and Mental Well-Being
Source: Nutrients. 2026 Apr 29;18(9):1405. doi: 10.3390/nu18091405 (PMC13165020; doi:10.3390/nu18091405)
Supplement: Supplementary file 1 [file nutrients-18-01405-s001.zip › nutrients-4241739-SI.pdf]

HOW TO DEVELOP A HEALTHY RELATIONSHIP WITH FOOD?

Balance begins within

In a society increasingly shaped by social media, our relationship with **food and our bodies** has become more complicated, as constant influences and expectations shape the way we perceive them. Contemporary literature emphasises that health is not just the mere absence of disease, but **complete physical, mental, and social well-being.** [1] Furthermore, growing research also suggests that overall well-being is closely linked to nutrition and mental health. [2]

This **guide** was created to support the development of **balanced**, sustainable **eating habits**, boost **motivation**, and strengthen the **connection** between **mental health, the body, and nutrition**.

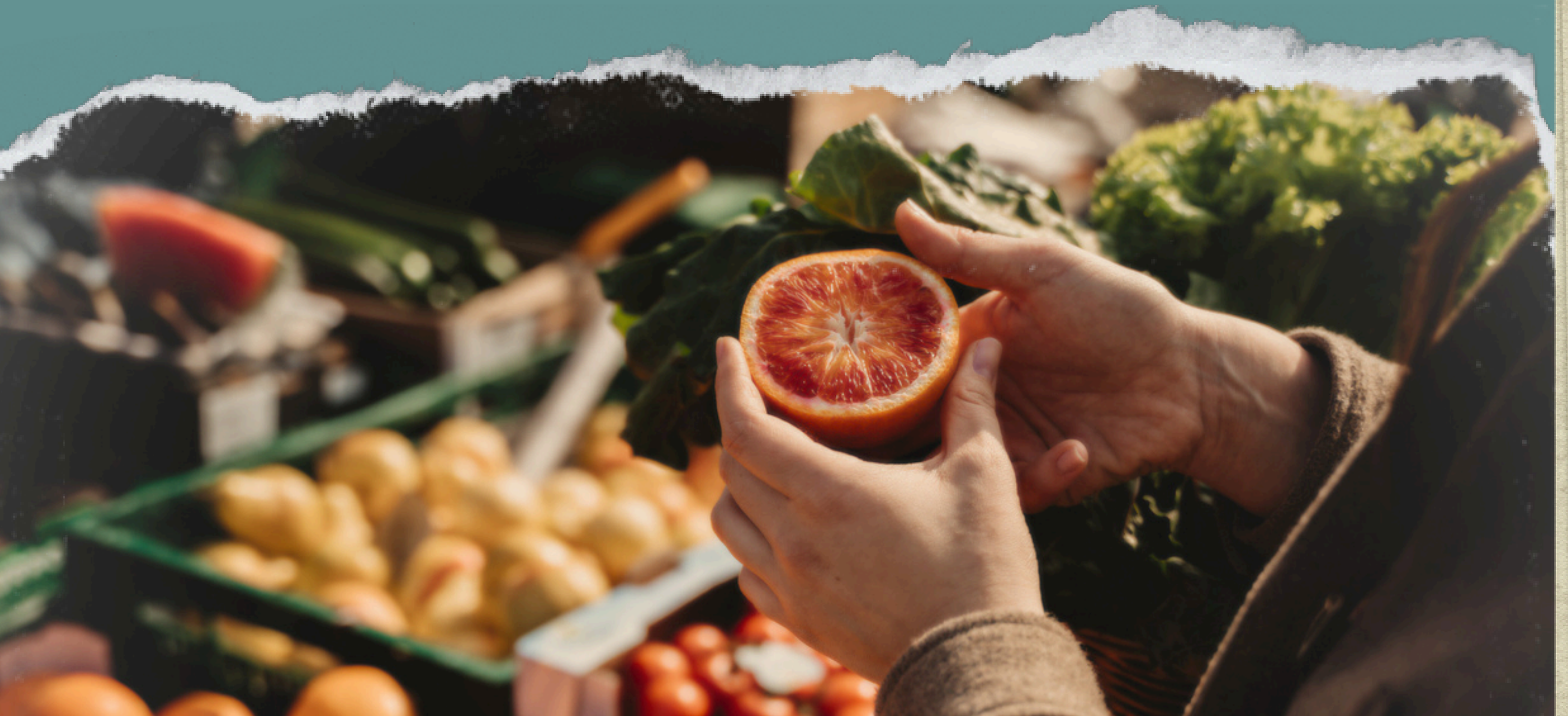

Discover your motivation
Why do you want to make a change?

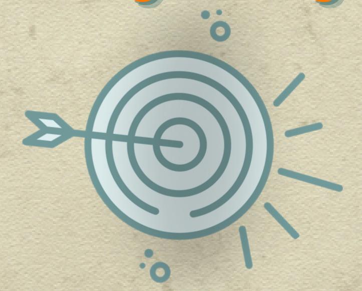

Genuine and sincere motivation is the driving force behind all lasting change. Without it, even the most meticulously planned diets can lead to disappointment and failure.

Research has shown that people with **internal motivation** (e.g., a desire for health, balance, energy, or personal growth) find it **easier to maintain healthy habits.**

In contrast, **external motivations**, such as social pressure, the desire for validation, or the pursuit of aesthetic standards, are often associated with **anxiety, low self-esteem, and the abandonment of the diet** [3–5].

Real and sustainable change occurs when we understand, accept, and **incorporate health recommendations into our personal value system**. When we follow these guidelines not because of external pressure, but because we are convinced of their importance they become an **integral part of our lifestyle** and essential steps toward lasting change. [4]

Contemplative exercise

Before you move on, take a few minutes for some **honest self-reflection**.

Answer the following questions:

- *Why do I want this change?*
- *What are the pros and cons of this change for me?*
- *Does my motivation stem from a desire for health and balance, or from the pressure to conform to norms?*
- *How would things change if I were to implement these changes?*
- *What would happen if I didn't change anything? [6]*

*List **three reasons** why you want to **eat more consciously.** Highlight the ones that are related to your **well-being**, not just your appearance. Consider whether you need to **adopt any of the suggestions** in the future.*

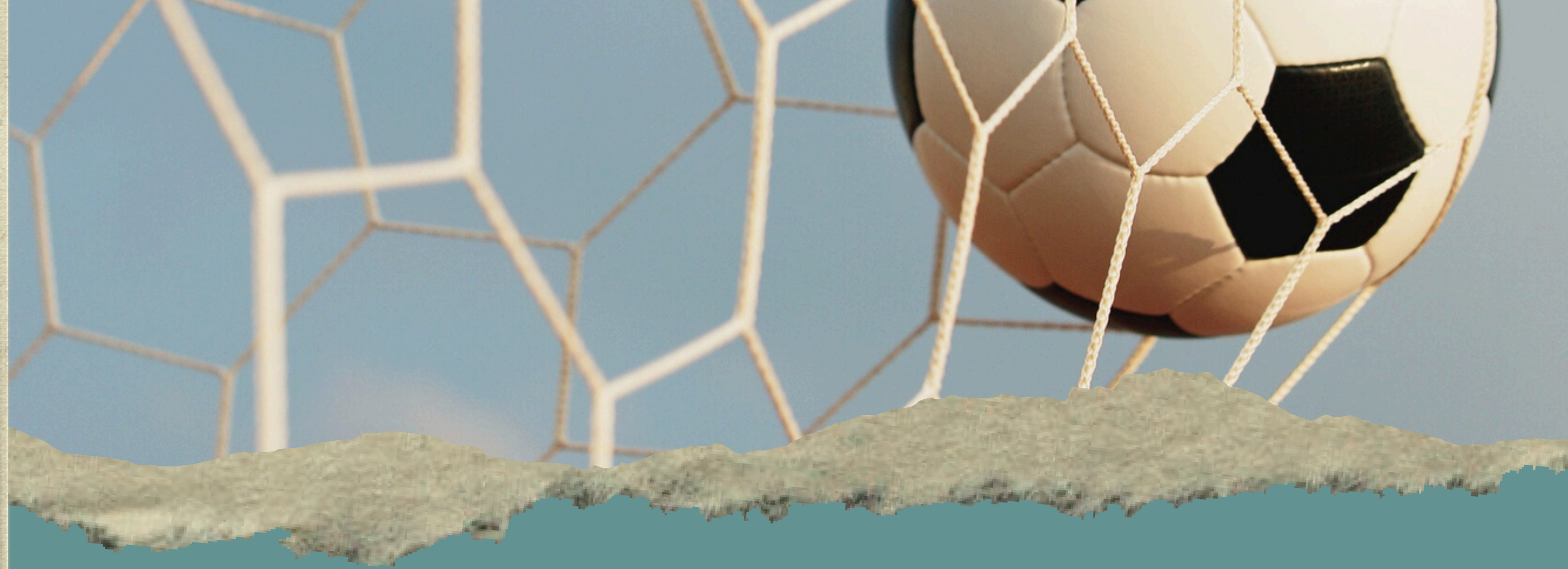

How can you set realistic goals?

To achieve sustainable change, it is worth applying the **START** method. [7]

S — **Specific:** Define exactly what you want to achieve.
"I want to lose 5 kg within 3 months through a balanced diet and regular exercise, which I will achieve by focusing on 30-minute workouts three times a week and planned meals."

T — **Time-bound:** Set a clear deadline that balances short- and long-term goals to stay motivated and avoid procrastination.
"I want to reach my goal within three months, losing about 0.5 kg per week."

A — **Aquisition:** How does a goal arise? It is important to clarify whether the goal arose from external pressure or from an internal decision.
"I set this goal for myself, and it stems from a sincere desire for health and balance."

R — **Rewards:** Establish a system for tracking progress. Monitoring progress and rewarding small achievements fosters commitment.
"I recognize milestones with symbolic rewards and adjust the plan as needed based on self-assessment."

T — **Tools:** List the tools that help you achieve your goal.
"I track my progress weekly using a food diary and an activity tracker app."

Write down your own START goal, then check to see if it meets all five criteria.

The basics of a balanced diet

There is no such thing as a perfect diet, only one that is tailored to the individual and meets their needs.

For a balanced diet:

- **Our diet should include all food groups.**

The body needs carbohydrates, protein, fats, vitamins, and minerals to function optimally. Completely eliminating certain food groups can lead to nutrient deficiencies and fatigue. [8]

Gyakorlati feladat:

Válassz ki egy ételt, *amelyet hajlamos lennél kerülni.* ***Készíts belőle kiegyensúlyozottabb változatot*** *úgy, hogy néhány hozzávalót táplálóbb alternatívára cserélsz, miközben a fogás élvezeti értéke megmarad.*

- **Avoid restrictions and the pursuit of perfection.**

Overly strict restrictions can lead to frustration and giving up. Flexible restrictions can result in more sustainable weight loss in the long run, as opposed to rigid control. [9]

- Find an **eating pattern** that you enjoy and can **stick to in the long run.**

- **Regular meals** and **adequate fluid intake** promote physical and mental balance.

- **Pay attention to your body's signals.**

If you get too hungry, you're more likely to make emotional food choices.

References:
1.Constitution of the World Health Organization. International Health Conference; 1946 Jul 22; New York, USA.
2. Himmerich H, Mirzaei K. Body Image, Nutrition, and Mental Health. Nutrients. 2024 Apr 10;16(8):1106.
3. Deci EL, Ryan RM. The "what" and "why" of goal pursuits: Human needs and the self-determination of behavior. Psychol Inq. 2000;11(4):227–68.
4. Ng JYY, Ntoumanis N, Thøgersen-Ntoumani C, Deci EL, et al. Self-determination theory applied to health contexts: A meta-analysis. Perspect Psychol Sci. 2012 Jul;7(4):325–40.
.5. Coumans JMJ, Lechner L, Bolman CAW, Oenema A. Clustering of motivational constructs based on self-determination theory for diet and physical activity and their associations with behaviour: a cross-sectional study. J Nutr Sci. 2022 Mar 17;11:e22. .

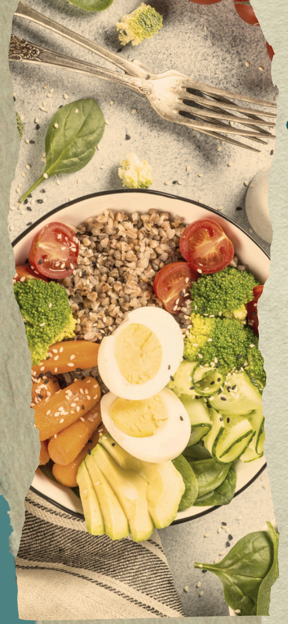

- **Practice mindful eating: [9]**
  - **Don't judge the experience of eating**. Don't attach negative or positive emotional connotations to foods; instead, maintain a balanced perspective and avoid thinking in terms of "good" or "bad" foods.
  - **Pay attention to colors, flavors, aromas, and textures.** This increases satisfaction and reduces mindless eating.
  - Focus on your food; **don't let anything distract you** (don't eat in front of a screen).
  - **Stop when you're full.** Learn to recognize when your body signals "enough."
  - **Recognize what triggers your food cravings**. Are you hungry, or are you just craving a particular food? Pay attention to your feelings without reacting impulsively to the craving.

It is important to distinguish between **physical and emotional hunger** (such as hunger caused by boredom, stress, or sadness): [10]

CHARACTERISTIC	PHYSICAL HUNGER	EMOTIONAL HUNGER
**Onset**	Gradually	Suddenly
**Desired meal**	Anything	Specific (sweet, salty)
**After eating**	Passes	Remains
**Feelings after eating**	Neutral	Guilt, shame

Keep a food diary for 3 days.
Note how you feel physically and emotionally before and after meals. Pay attention to how much you actually eat, and whether there are times when you reach for food for reasons other than physical hunger.

6. Barley E, Lawson V. Using health psychology to help patients: theories of behaviour change. Br J Nurs. 2016 Sep 8;25(16):924–
7.Pearson ES. Goal setting as a health behavior change strategy in overweight and obese adults: a systematic literature review examining intervention components. Patient Educ Couns. 2012 Apr;87(1):32–42.
8. Kiani AK, Dhuli K, Donato K, et al. Main nutritional deficiencies. J Prev Med Hyg. 2022 Oct 17;63(2 Suppl 3):E93–E101.
9. Dohle S, Diel K, Hofmann W. Executive functions and the self-regulation of eating behavior: A review. Appetite. 2018 May 1;124:4–9.

Body Image and Self-Acceptance

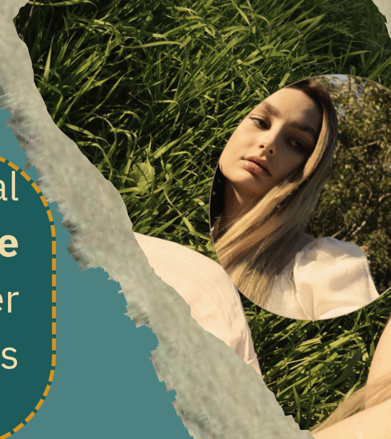

The concept of **body image** describes how a individual perceives, feels, and evaluates their own body. A **positive** body image does not mean perfection, but rather **acceptance and respect** for the body, as well as **appreciation** for what it enables them to do every day. [11]

People who express **gratitude** toward their bodies tend to be more emotionally balanced and find it **easier to develop healthy eating habits.** Gratitude toward the body involves recognizing and **appreciating everything the body does,** beyond physical appearance. [12]

Write down one thing each day that you are grateful to your body for (e.g., "I walked more today and felt energetic.").

Self-affirmation refers to the process through which an individual reflects on their own personal values. This helps the brain respond more effectively to threats and criticism, thereby **reducing defensive reactions.** [13]

Write a short paragraph about one or two values that are particularly important to you (e.g., family, humor, kindness, or religion). Explain why these values are meaningful to you, and describe a situation in which you acted in accordance with them.

Expressing **gratitude** toward the body and practicing **self-affirmation** can enhance stress management, **reduce the risk of disordered eating patterns, improve self-confidence and body image,** and foster a more balanced perspective on health and everyday decisions. [12, 13]

This material was developed for ***educational purposes*** *to promote a healthy relationship between nutrition, mental well-being, and body image. The content is based on* ***scientifically grounded*** *principles and professional recommendations from the fields of* ***psychology*** *and* ***nutrition science.***

10. Peitz D, Schulze J, Warschburger P. Getting a deeper understanding of mindfulness in the context of eating behavior: Development and validation of the Mindful Eating Inventory. Appetite. 2021 Apr 1;159:105039.
11. Tylka TL, Wood-Barcalow NL. The Body Appreciation Scale-2: Item refinement and psychometric evaluation. Body Image. 2015 Jan;12:53–67.
12. Homan KJ, Tylka TL. Development and exploration of the gratitude model of body appreciation in women. Body Image. 2018 Jun;25:14–22..
13.Cohen GL, Sherman DK. The psychology of change: Self-affirmation and social psychological intervention. Annu Rev Psychol. 2014;65:333–71.
